# Physiological Differences Underlying Divergent Hypoxia Responses and Altitude Adaptations in Humans, Rats and Mice

**DOI:** 10.1002/cph4.70077

**Published:** 2025-11-27

**Authors:** Johannes Burtscher, Robert T. Mallet, Anupam Sah, Max Gassmann, Martin Burtscher, Rodrigo Iturriaga

**Affiliations:** ^1^ Institute of Sport Science University of Innsbruck Innsbruck Austria; ^2^ Department of Psychiatry, Psychotherapy, Psychosomatics and Medical Psychology, University Hospital for Psychiatry II Medical University of Innsbruck Innsbruck Austria; ^3^ Department of Physiology and Anatomy University of North Texas Health Science Center Fort Worth Texas USA; ^4^ Institute of Veterinary Physiology, Vetsuisse Faculty University of Zurich Zurich Switzerland; ^5^ The Zurich Center for Integrative Human Physiology (ZIHP) University of Zurich Zurich Switzerland; ^6^ Universidad Peruana Cayetano Heredia Lima Peru; ^7^ Instituto de Ciencias Biomédicas Universidad Autónoma de Chile Santiago Chile

**Keywords:** chronic continuous hypoxia, erythropoietin, high altitude adaptation, human physiology, hypoxia inducible factor, intermittent hypoxia conditioning, mouse, rat, red blood cells, species differences

## Abstract

Hypobaric hypoxia, a defining feature of high‐altitude environments, poses a considerable physiological challenge to both humans and rodents. To withstand hypoxic stress, mammals have developed cellular and systemic adaptations that not only safeguard against acute and future episodes of oxygen deprivation but may also enhance overall resilience and functional capacity. A central aim of current research is to harness these health‐promoting effects of hypoxic exposure as a therapeutic strategy for a range of medical conditions. To date, much of the evidence regarding the safety and efficacy of such interventions derives from rodent studies. In this review, we summarize current knowledge on hypoxia tolerance, oxygen transport, and oxygen consumption in humans, rats, and mice, and evaluate the extent to which findings from rodent models can be extrapolated to humans. While the anatomical, physiological, and molecular foundations of oxygen transport and utilization are broadly conserved across species, there are important quantitative differences—largely linked to body‐mass variation—as well as qualitative distinctions. Mice that evolved in high‐altitude environments, display remarkable hypoxia tolerance. Their physiological repertoire includes highly efficient pulmonary gas exchange, metabolic downregulation, and substantial plasticity of the mitochondrial electron transport system under hypoxic conditions. In contrast, rats exhibit heightened vulnerability in hypoxia, manifesting as right ventricular hypertrophy, excessive erythropoiesis, and myocardial injury. These interspecies differences highlight that the robust hypoxia tolerance of mice—and the potentially comparatively greater susceptibility of rats than humans—must be carefully considered when translating findings from rodent hypoxia research into human contexts.

## Graphical abstract

Despite many physiological similarities, mice, rats and humans respond remarkably differently to hypoxia. In line with their high‐altitude phylogeographic history, mice are physiologically well equipped to deal better with high‐altitude hypoxia than humans, while the low‐land origin of rats may be associated with a particularly high vulnerability to hypoxia.

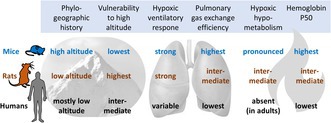

## 1 Background

An increasing body of research evidence demonstrates the health‐promoting potentials of controlled exposure to hypoxia, that is, low oxygen availability (Ehrenreich et al. 2023; Burtscher, Citherlet, et al. 2024; Rogers and Mootha 2025). The possibility that prolonged ambient or inspiratory hypoxia may attenuate oxidative stress and promote health, healthy aging and/or longevity is currently intensely discussed (Rogers and Mootha 2025). The hypothesis that physiological responses elicited by hypoxia induce health benefits is supported by empirical evidence that hypoxia promotes favorable cellular and systemic adaptations. Theoretically, such adaptations may increase resistance to hypoxic insults, augment cellular metabolism and resilience, and bolster systemic oxygen transport. Approaches to elicit such hypoxia responses, hypoxia conditioning programs (Burtscher, Citherlet, et al. 2024), include intermittent or transient exposures to ambient or inspiratory hypoxia (e.g., acute intermittent hypoxia conditioning, intermittent hypoxia training/therapy/exposure/conditioning). Importantly, the severity, duration and frequency of these therapeutic hypoxia exposures are carefully calibrated to avoid harm, unlike pathological chronic intermittent hypoxia, as occurring for example in obstructive sleep apnea (OSA). The key distinctions between therapeutic and harmful intermittent hypoxia have been reviewed comprehensively (Navarrete‐Opazo and Mitchell 2014). Due to the assumed relative safety of these approaches, they have been applied in humans for decades. Publications on clinical and ergogenic applications of these methods have surged recently. Despite unclear evidence for many applications and frequent terminological confusion, many commercial suppliers of hypoxia equipment have appeared, serving a poorly regulated market (Panza et al. 2023). Some of the claims of this emerging industry are based largely on the results of rodent studies and include the tempting assertions, not yet established in humans, that intermittent hypoxia conditioning improves mitochondrial functions (e.g., by inducing mitophagy), promotes the utilization of fat and reduces inflammation.

Studies in cells and some invertebrate and vertebrate species have demonstrated that either environmental hypoxia or the reduction of non‐physiologically high oxygen levels (hyperoxia) in cell culture—prolong lifespan or viability (Packer and Fuehr 1977; Rascón and Harrison 2010; Rogers et al. 2023). Although highly interesting for humans, these results must be evaluated critically, especially when solely considering cell culture experiments. Supraphysiological oxygen levels, often termed ‘normoxic’, but in reality hyperoxic, typify cell culture conditions (Prabhakar and Semenza 2012; Wenger et al. 2015). This hyperoxia has profound implications for cell biology, including aging. Culturing cells at supraphysiological oxygen concentrations influences diverse cellular processes, such as redox regulation and cellular respiration, gene expression, replicative lifespan, migration and differentiation (Alva et al. 2022). For example, growing mouse embryoid bodies (a model of embryogenesis) under chronically hyperoxic conditions (20% oxygen), impaired hemoglobin differentiation (Bichet et al. 1999). The physiological oxygen conditions of different cell types in many organs have been extensively reviewed elsewhere, allowing determination of appropriate oxygen concentration for culturing different cell types (Keeley and Mann 2019).

Findings in animal models led to the hypothesis that impaired oxygen utilization (e.g., due to mitochondrial dysfunction) can cause localized hyperoxia (Rogers and Mootha 2025) which may elicit excessive formation of reactive oxygen intermediates and, thereby, impose oxidative stress. On the other hand, genetically or chemically inhibiting mitochondrial components to reduce cellular respiration increased the lifespan of cultured cells (Packer and Fuehr 1977), worms (Feng et al. 2001; Lee et al. 2003; Kayser et al. 2004; Mehta et al. 2009) flies (Copeland et al. 2009) and mice (Dell'agnello et al. 2007; Lapointe and Hekimi 2008), indicating that lowering oxygen utilization may improve aging, possibly also through reduced oxidative stress. More recently, substantial benefits of chronic continuous hypoxia exposure have been demonstrated in mouse models of various diseases involving mitochondrial dysfunction, including multiple sclerosis, Leigh syndrome, Friedreich's ataxia (recently reviewed (Burtscher, Motl, et al. 2025; Rogers and Mootha 2025)) and Parkinson's disease (Marutani et al. 2025). In addition, permanent hypoxia increased life span in a mouse model of accelerated aging (Rogers et al. 2023). These studies typically expose mice to atmospheres containing about 11% oxygen (vs. 21% oxygen in normoxic air) corresponding to terrestrial altitudes ≥ 5000 m. These severely hypoxic conditions are considered borderline for human adaptation and permanent residence (Champigneulle et al. 2023) but are considered “mild hypoxia”, because the mice tolerate them well. The question therefore arises, to what degree the important benefits of chronic hypoxia exposure, as well as of intermittent hypoxia conditioning, can be translated from mice to humans.

Here, we focus on crucial physiological differences related directly or indirectly to hypoxia responses/tolerance in humans versus rats and mice, the most commonly studied animal models in hypoxia research. The implications of the divergent hypoxia physiology of rodents versus humans for pathological processes related to severe hypoxia and for therapeutic hypoxia applications are discussed.

## 2 Pharmacological Modulation of Hypoxia Inducible Factor Pathways

Aside from the direct application of controlled hypoxia, the molecular machinery of responses to hypoxia has been targeted to modulate health outcomes. Changes in oxygen levels, in particular when hypoxia is alternated with reoxygenation, can elicit energetic depletion, oxidative stress, and immune and inflammatory responses (Prabhakar and Semenza 2012; Burtscher, Mallet, et al. 2022; Iturriaga and Diaz 2025). Pivotal molecular pathways quickly respond to altered oxygen availability (Lee et al. 2020), including the transcription factors hypoxia‐inducible factors (HIFs), of which HIF‐1 and HIF‐2 are considered the primary coordinators of cellular hypoxia responses. HIF pathways strongly interact with many other pathways related to cellular energy (e.g., AMP‐activated protein kinase [AMPK]), oxidative stress (e.g., nuclear factor erythroid 2‐related factor 2 [Nrf2]) or inflammation (e.g., nuclear factor kappa‐light‐chain‐enhancer of activated B cells [NF‐κB]). The resultant cellular hypoxia responses are geared toward counteracting energetic crises, oxidative stress, excessive inflammation, cell death and tissue damage. HIF pathways also mediate medium‐ to long‐term plastic changes of the respiratory system during prolonged hypoxia (Prabhakar and Semenza 2012).

**FIGURE 1:**
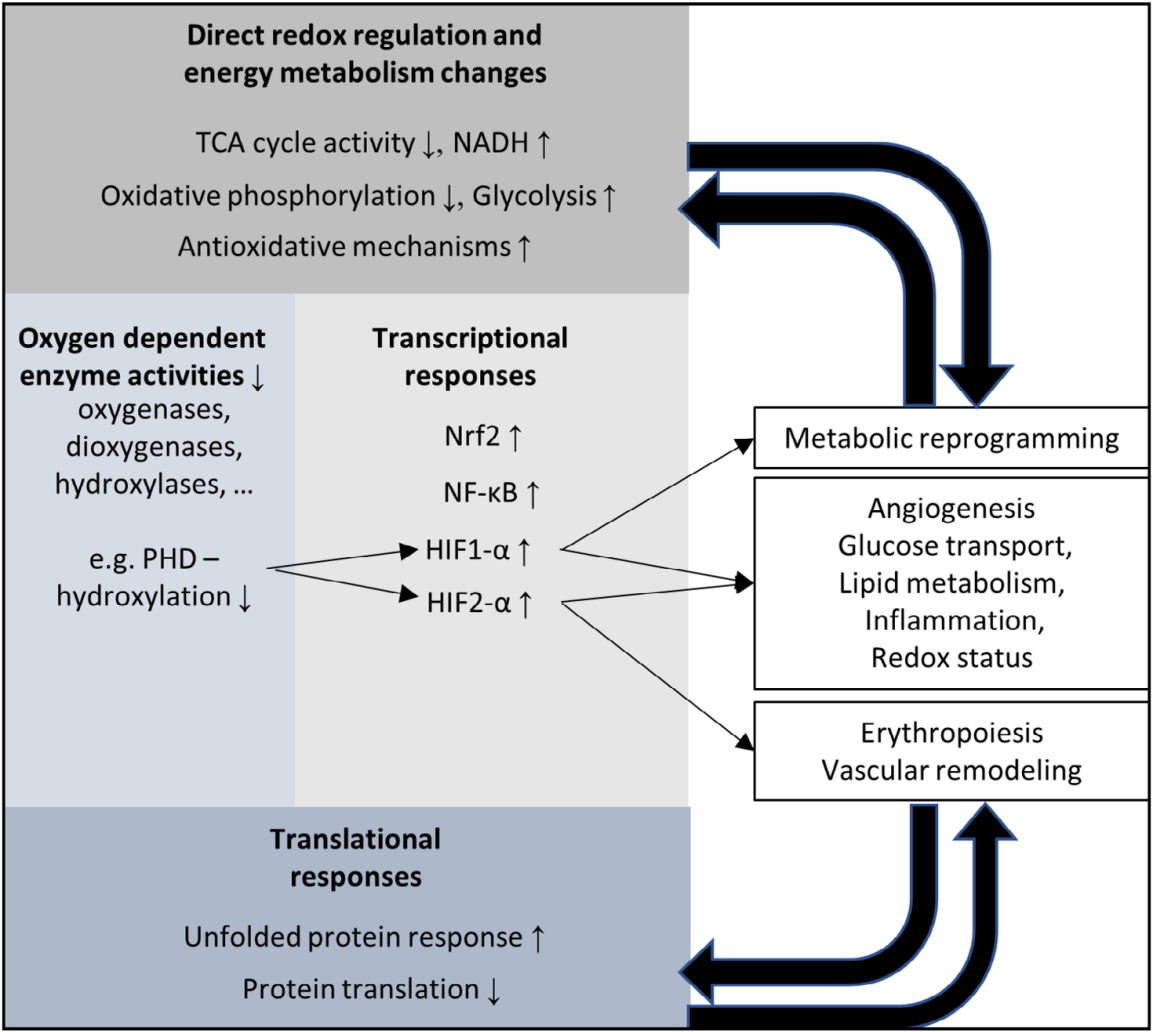
Cellular hypoxia responses. HIFs, hypoxia inducible factors; NADH, reduced nicotinamide adenine dinucleotide; NF‐κB, nuclear factor kappa‐light‐chain‐enhancer of activated B cells; Nrf2, nuclear factor erythroid 2‐related factor 2; PHD, prolyl‐4‐hydroxylase domain containing proteins; TCA, tricarboxylic acid cycle.

The genetic or pharmacological down‐ or upregulation of components of hypoxic response pathways, particularly those elements affecting HIF‐1 and HIF‐2‐related processes, is a rapidly expanding research field, involving prominent applications, for example, for cancer and chronic kidney disease (CKD) therapies (Semenza 2019; Stoumpos et al. 2024). HIF pathways are also potential treatment targets for neurological disorders, including stroke (Pan et al. 2021), Alzheimer's disease (Liu et al. 2023) or Parkinson's disease (Burtscher, Duderstadt, et al. 2024). HIF‐1 and HIF‐2 are hetero‐dimeric transcription factors that are sensitive to changes in cellular oxygen levels. During normoxia, the HIF‐1α and HIF‐2α subunits are hydroxylated by HIF prolyl hydroxylases (PHDs) (Kaelin and Ratcliffe 2008) and, thereby, directed toward poly‐ubiquitinylation and proteasomal degradation to suppress hypoxia‐responsive gene expression. Hypoxia opposes hydroxylation and subsequent degradation of HIF α subunits, allowing them to dimerize with HIF β subunits (also termed Aryl Hydrocarbon Receptor Nuclear Translocator; ARNT), forming a complex that regulates the transcription of hundreds of genes involved in the hypoxia response (Burtscher, Mallet, et al. 2022). While HIF‐1α and HIF‐2α are regulated similarly by PHDs and hypoxia, they are differentially expressed spatially (HIF‐1 is expressed ubiquitously, HIF‐2 is restricted to certain tissues (Prabhakar and Semenza 2012)) and temporally (HIF‐1 is induced more rapidly, HIF‐2 remains stable longer (Koh and Powis 2012)). Cell‐culture experiments suggest that HIF‐1α levels peak within 24 h after exposure to a sufficient hypoxic dose and decline toward baseline thereafter, while HIF‐2α stays upregulated for days (Koh and Powis 2012). Moreover, certain molecular regulators, for example, factor inhibiting HIF‐1 (FIH), preferentially control either HIF‐1 or HIF‐2, imparting specificity to clinically relevant HIF stabilizers (e.g., PHD inhibitors) that do not inhibit FIH. Consequently, the FIH‐inert PHD inhibitors may stabilize HIF‐2 more effectively than HIF‐1 (Semenza 2019) and thus, selectively induce HIF‐2‐activated pathways, such as the erythropoietin‐erythroferron‐hepcidin pathway (Gassmann and Muckenthaler 2015). Moreover, in contrast to HIF‐1, HIF‐2 is not expressed in invertebrates (Prabhakar and Semenza 2012). Taken together, HIF‐1 and HIF‐2 regulate distinct (but also overlapping) molecular processes (Burtscher et al. 2023). Apart from transcriptional responses, a downregulation of energy‐intensive cellular processes occurs, encompassing for example reduced translation of RNA into proteins (Lee et al. 2020). Figure 1 summarizes important cellular hypoxia responses.

In CKD‐related anemia, the efficacy of HIF stabilization by inhibiting the negative HIF regulators PHDs has been investigated in dozens of phase 3 clinical trials (Stoumpos et al. 2024) and some molecules already have been approved for marketing in the United States (daprodustat and vadadustat) (Haase et al. 2024) or other countries (e.g., roxudastat) (Wang et al. 2022).

In summary, the pharmacological modulation of HIF pathways is a promising avenue to treat diseases linked to hypoxia or dysregulated hypoxia responses. Especially the use of PHD inhibitors is currently being thoroughly scrutinized for long‐term and potential adverse effects, which may include adverse effects on cancer growth and other problematic unwanted on‐ and off‐target effects (Stoumpos et al. 2024). The extensive literature on preclinical and clinical (including a large number of phase 3 clinical trials) studies of pharmacological modulation of HIF pathways is contrasted by comparably small, heterogeneous and difficult‐to‐compare pieces of evidence from studies on health effects of controlled hypoxia exposures, the main focus of the present review. The progress on therapeutic benefits of pharmacological upregulation of molecular hypoxia response pathways in humans supports the idea of health promotion through induction of such pathways by controlled ambient/inspiratory hypoxia. However, the assessment of safety and efficacy of such interventions is complicated by uncertainty about the translational validity for human applications from the results of rodent experiments, from which most of the preclinical evidence has been obtained. However, different hypoxia responses and adaptations in rodents, call into question the translational validity of those studies. The comparison of naturally occurring high‐altitude populations of humans and rodents may yield crucial insights regarding potential species differences in their adaptive capacities to altitude and hypoxia, vulnerabilities, as well as the efficacy of therapeutic hypoxia or hypoxia‐related medications.

## 3 Humans and Rodents Living in Altitude Environments; Tolerance and Mortality

Mammals, including humans and rodents, have populated high (2500–3500 m) and some species even very high (3500–5500 m) altitude regions, despite hypobaric hypoxia and other challenges. In the wild, mouse populations inhabit altitudes far above 4000 m. Recently, a wild yellow‐rumped leaf‐eared mouse (*Phyllotis xanthopygus rupestris*) was discovered at the 6739 m summit of Llullaillaco (Storz et al. 2020). Rats do not live at such high altitudes (Storz et al. 2007; Jochmans‐Lemoine et al. 2015). Indeed, rats and mice have very different phylogeographical histories. The house mouse ( *Mus musculus* ) originated from the Himalayan region, from where it colonized other Eurasian territories via migration that included traversing extensive mountain chains (Bonhomme and Searle 2012; Arias‐Reyes et al. 2021). The migrating mice thereby likely underwent natural selection that conferred a “pre‐adaptation” to high altitude environments (Arias‐Reyes et al. 2021). In contrast, common rats ( *Rattus rattus* and *Rattus norvegicus* ) originated from Chinese and Indian lowlands, from where they migrated through lowlands to other regions, strongly linked to human migratory routes (Aplin et al. 2011; Puckett et al. 2016).

The highest permanently inhabited city is La Rinconada, located in Peru at 5000–5300 m. High average hemoglobin concentrations and a high prevalence of chronic mountain sickness underscore the challenges of human habitation at such altitudes (Champigneulle et al. 2023), especially for lowlanders translocating to high altitude, although efficient transgenerational adaptations exist in highland populations (Santolaya et al. 1989; Bigham and Lee 2014). While the role of continuous moderate ambient hypoxia exposure (e.g., at 1000–2500 m altitude) on human health remains poorly understood, several observational data suggest that moderate and even high‐altitude environments may also be beneficial for human health (Burtscher and Samaja 2024; Burtscher, Kopp, et al. 2025). Table 1 shows a commonly used categorization of altitude categories.

**TABLE 1:** Altitude levels and characterization (approximate values).

Altitude category (m)	Barometric pressure (mmHg)	Inspired partial pressure of oxygen (PO_2_) in %	Inspired fraction of oxygen (F_I_O_2_) × 100 (%)
Low (0–1000)	760–680	150–130	20.9–18.4
Moderate (> 1000–2500)	< 680–560	< 130–110	< 18.4–15.3
High (> 2500–3500)	< 560–470	< 110–95	< 15.3–13.5
Very high (> 3500–5500)	< 470–380	< 95–70	< 13.5–10.5
Extreme (> 5500)	< 380	< 70	< 10.5

Notably, moderate and high‐altitude environments are not only characterized by hypoxia, but also by other geoclimatic and socioeconomic peculiarities. In addition, controlling for ethnic and behavioral differences of populations living at different altitudes often is difficult; thus, a substantial number of potentially confounding factors must be considered in human altitude research. Still, altitude‐dependent changes in the partial pressure of atmospheric oxygen (PO_2_), that is, hypobaric hypoxia, are pivotal physiological factors that become more prominent with increasing altitude and, thus, decreasing PO_2_. Hypoxia is therefore assumed to contribute to the reduced morbidity and mortality from different causes reported at moderate altitude as compared to low altitude (Burtscher, Kopp, et al. 2025; Burtscher, Strasser, et al. 2025). Lower mortality from cardiovascular and metabolic diseases and certain cancers appears to be the primary reason for reduced overall mortality at moderate altitude, as observed for example in populations in the United States for cardiovascular diseases (Mortimer Jr et al. 1977; Ezzati et al. 2012), cancers (Youk et al. 2012) or dementia (Thielke et al. 2015). Similarly, reduced all‐cause mortality (Burtscher et al. 2021; Burtscher, Strasser, et al. 2025), mortality from cardiovascular diseases and stroke (Faeh et al. 2009; Burtscher et al. 2021; Burtscher, Strasser, et al. 2025) and from cancers like colorectal and breast cancer (Burtscher et al. 2021), were reported in European populations residing at moderate altitudes. Moreover, mortality advantages have been reported in high‐altitude dwellers in South America; for example reduced ischemic heart disease rates at altitudes above 2500 m (Ortiz‐Prado et al. 2023) and lower mortality from stroke above 1500 m (Ortiz‐Prado et al. 2021) in Ecuador.

Overall, human mortality benefits appear to mainly apply to residence at moderate altitude. In contrast, altitudes above 2000 m may favor cancer growth, at least in an Ecuadorian population (Garrido and Garrido 2018) and high altitudes above 2500 m may lead to other diseases, including chronic mountain sickness. Importantly, high‐altitude adaptations are strongly modulated by ethnic factors and several underlying genetic differences have been described that modulate high‐altitude tolerance. In contrast to immediate physiological and pathophysiological hypoxia responses and adaptations to acute or prolonged hypoxia (sometimes termed plasticity to distinguish from genetic adaptation), transgenerational genetic adaptations frequently occur at the population level at high altitude and can facilitate life there. These genetic adaptations have been reported in humans and in other mammals and have been the focus of recent reviews (Pamenter et al. 2020; Storz and Cheviron 2021; Lee 2024). Despite specific variants, many of the genetic high‐altitude adaptations of different species converge on similar genes, especially those related to the PHD‐HIF axis (Pamenter et al. 2020).

Among the most famous of such genetic adaptations are polymorphisms described in human Tibetan highlanders in *EPAS1* and *EGLN1*, coding for HIF‐2α and PHD2, respectively. Both mutation types have been associated with a lack of excessive erythrocytosis, persistently increased hypoxic ventilatory responses (HVRs) and lower right ventricular pressures (Lee 2024). Conversely, ARNT2 (but not ARNT), which can heterodimerize with HIF‐2α has been suggested to be a target of genetic selection for high altitude life in Ethiopians (Scheinfeldt et al. 2012). Yet other mechanisms have been reported for Andean highlanders, who show relatively strong increases in hemoglobin concentrations with increasing altitude and are at greater risk of developing chronic altitude sickness, as compared to Tibetans, who have lower hemoglobin concentrations at high altitudes than most other ethnic groups (Bigham and Lee 2014; Gassmann et al. 2019), and are relatively protected from chronic mountain sickness.

Genetic adaptations related to the HIF pathway that may confer improved tolerance to altitude have been described in other mammalian species, including deer mice, cattle, goats, boars, horses, dogs and snow leopards (recently reviewed in Lee 2024). However, the naturally occurring genetic adaptations to altitude, for example of HIF‐2α, in mice are different from those of humans. This is the case in the high‐altitude North American deer mouse, which developed hemoglobin with an increased affinity for oxygen (Natarajan et al. 2013) and a reduced ventilatory chemosensitivity. These adaptations, however, do not alter hematological parameters (Ivy et al. 2022).

To investigate inter‐species differences in hypoxia adaptations, laboratory rats (Sprague Dawley) and mice (genetic mix of the strain FVB and others) raised for > 30 generations at 3600 m (La Paz, Bolivia) were studied (Jochmans‐Lemoine et al. 2015). At baseline conditions (21% oxygen, at 3600 m with a mean barometric pressure of about 490 mmHg), these mice and rats had similar pO_2_ and oxygen saturation. Conversely, hematocrit and blood hemoglobin content were lower in mice. Right ventricular hypertrophy was less severe in the mice (indicating milder pulmonary hypertension), while mass‐corrected lung volume, alveolar surface area, minute ventilation (only in females), tidal volume, heart rate and metabolic rate were higher in mice. However, the mice had a lower respiratory exchange ratio (oxygen consumption rate/CO_2_ production rate). Moreover, mice inspired significantly more oxygen and expired more CO_2_, both absolute and mass‐corrected. During brief (10 min) exposures to moderate hypoxia (18% oxygen), mice better preserved oxygen consumption, and—as opposed to the human hypoxia response, and unlike rats—showed a significantly reduced heart rate and tidal volume, without notable changes in ventilation (this “hypoxic hypometabolism” phenomenon is discussed in more detail in Section 4.5). Further reduction of the ambient oxygen concentration to 15% (corresponding to a partial pressure of inspired O_2_ of about 75 mmHg) for 10 min intriguingly abolished most of the physiological divergences between mice and rats, except for the metabolic rate (both oxygen consumption and CO_2_ production) (Jochmans‐Lemoine et al. 2015). The metabolic rate was significantly reduced compared to baseline in mice, but not in rats, already at a partial pressure of inspired O_2_ of about 90 mmHg and even more at 75 mmHg. Overall, right ventricular hypertrophy and excessive erythropoiesis, signs of chronic mountain sickness, were present in rats, but not in mice, living since generations at 3600 m. Higher mass‐corrected lung volume and alveolar exchange surface area may be anatomical protective factors in mice, while the metabolic responses (reduced metabolic rate and heart rate) appear to be protective physiological mechanisms.

In summary, mice are more tolerant to hypoxia than humans and rats are. Humans and mice share similar genetic high‐altitude adaptations which can result in distinct phenotypes. In humans, moderate altitude has been shown to be associated with lower mortality from various causes across different populations. However, higher altitudes are more difficult for humans to tolerate and increasingly lead to health challenges that appear to constitute a barrier to populating altitudes above around 5000 m. Besides long‐term altitude acclimatization (including high‐altitude residence) and genetic adaptations, acute physiological responses and adaptations to altitude exposures are important strategies to survive hypoxia conditions. The following section discusses differences in these mechanisms among humans, rats and mice.

## 4 Physiological Inter‐Species Differences Related to Oxygen Transport and Hypoxia

Rodent models are and have been invaluable tools for the investigation of human diseases. The anatomic, physiological and genetic similarities of rodents to humans (especially as compared to non‐mammalian organisms and in vitro systems), combined with the short generation time and the available tools for genetic and toxicological/pharmacological manipulation make rodents particularly useful model organisms (Vandamme 2015; Götz et al. 2018). However, rodents are not humans, and the diseases modeled in them usually do not fully reflect the human counterparts. The validity of many rodent models is for example particularly questionable for neurodegenerative diseases, like Alzheimer's disease (Götz et al. 2018) or Parkinson's disease (Vingill et al. 2018). The long human lifespan and the unique architecture and complexity of the human brain (Kanari et al. 2025) play important roles in the pathogenesis. Consequently, very few novel therapies for neurodegenerative diseases have been successfully translated from rodent models to clinics. For treatments with hypoxia, several additional differences between human and rodent (and also among rodent species) physiological responses must be considered and are described in this section.

### 4.1 Cell Biology and Molecular Stress Pathways

Despite overall relatively conserved cell‐biological and physiological responses to cellular stress, several stress responses differ between humans, rats and mice. Inflammatory and immune processes, for example, are notoriously different in mice versus humans (Seok et al. 2013), although other researchers report similar responses in both species (Takao and Miyakawa 2015). Species differences may especially concern neuroinflammation, for example since human microglia, the brain's resident immune cells, show signs of moderate activation even in health, which is not the case in mice (Lassmann 2020).

Oxidative stress responses may diverge among humans and rodents as well, since more long‐lived mammals tend to be characterized by greater accumulation in mitochondrial DNA of the oxidative damage marker 8‐hydroxy‐2′‐deoxyguanosine than shorter‐lived mammals (Barja 2002). During immune responses, however, human immune cells produce more reactive oxygen species (ROS) as compared to rodents, where the production of nitric oxide seems to be favored in certain conditions (Wink et al. 2011). Redox regulation in hypoxia differs between mice and rats. Comparison of the activities of antioxidant enzymes and of mitochondrial cytochrome oxidase‐c in lungs of mice and rats, raised at either sea level (Jochmans‐Lemoine et al. 2016) or 3600 m (Jochmans‐Lemoine et al. 2015) revealed only small differences between low‐ and high‐altitude mice (Jochmans‐Lemoine et al. 2018). However, significantly increased cytochrome oxidase‐c (2‐fold) and mitochondrial antioxidant enzyme (superoxide dismutase and glutathione peroxidase) activities were observed in lung mitochondria of high‐ versus low‐altitude rats (Jochmans‐Lemoine et al. 2018). The robust induction of pulmonary mitochondrial antioxidant enzymes in high‐ versus low‐altitude rats may represent compensation for the potentially pathophysiological mitochondrial responses to altitude in rats versus the more hypoxia‐adapted mice.

Exposure of FVB‐NJ mice and Sprague–Dawley rats to 12% oxygen, 6 h/day, for 21 days increased mitochondrial oxygen consumption rate in liver mitochondria in mice but not in rats (Arias‐Reyes et al. 2021). This effect was mainly related to the plasticity of murine mitochondrial complex II and was associated with significantly greater ventilation and mass‐normalized whole‐body oxygen consumption as compared to rats.

Taken together, cellular responses between humans, rats and mice can differ in redox regulation, inflammatory responses and energy metabolism plasticity. Regarding hypoxia responses, the mitochondrial plasticity of mice seems to be a protective factor, while excessive upregulation of antioxidant enzymes may be a maladaptive mechanism in rats.

### 4.2 HIF Pathways and Transcriptional Responses to Hypoxia

While HIF pathways are central to the hypoxia responses of humans, rats and mice, their activities differ quantitatively and qualitatively. A meta‐analysis comparing the transcriptomes in response to hypoxia in humans and mice observed substantial overlap in the genes regulated by HIFs in the 2 species, but also notable differences, including genes related to redox balance and energy metabolism (Bono and Hirota 2020). For example, the gene encoding neuron‐specific enolase was upregulated in almost all human transcriptome datasets after hypoxia, but was not hypoxia‐responsive in mouse transcriptomes (Bono and Hirota 2020). Hypoxic induction of HIF‐1 expression differed appreciably in the brains of mice and rats. While in mice hypobaric hypoxia (0.4 ATM, corresponding to about 7000 m altitude) sharply increased HIF‐1α content within about 4–7 h before a gradual decline (Benderro and LaManna 2014), HIF‐1α accumulation in rat neurons after 6 h exposure to 10% oxygen (corresponding to about 6000 m altitude) was modest and restricted to some brainstem circuits related to cardiorespiratory hypoxia responses (Pascual et al. 2001). Confirming these results, the physiological hypoxia responses in mice (FVB strain) raised at sea level were associated with a significant and dose‐dependent increase of HIF‐1α expression in the brainstem (Jochmans‐Lemoine et al. 2016). This response was absent in Sprague Dawley rats raised at sea level during 6 h exposures to 15% and 12% oxygen (corresponding to about 2500 m and 4500 m, respectively). The robust HIF response in mice was correlated with stronger increases of mass‐corrected ventilation and reductions of oxygen consumption and CO_2_ production during hypoxia, compared to the HIF response in the brainstem of hypoxic rats.

The gene expression in the lung was compared in Sprague–Dawley rats and C57BL/6 mice following 1 or 3 weeks of hypobaric hypoxia (about 5200 m) (Hoshikawa et al. 2003). Distinct transcriptional responses included upregulation of genes involved in endothelial cell proliferation and vasodilation in rats versus downregulation of those genes in mice (Hoshikawa et al. 2003). These divergent responses were associated with reduced pulmonary vascular wall thickening in mice as compared to rats (Hoshikawa et al. 2003), which may reflect greater hypoxia tolerance in mice.

The collective evidence demonstrates more robust HIF responses in mice than in rats. In contrast, potentially detrimental upregulation of genes involved in vascular remodeling was apparent only in rats.

### 4.3 Species Differences in Oxygen Chemosensing

The systemic responses to hypoxia in humans have been well investigated and reviewed (Teppema and Dahan 2010; Burtscher, Niedermeier, et al. 2022; Luks and Hackett 2022). In brief, a low arterial partial pressure of oxygen (PaO_2_) is sensed by peripheral chemosensors, primarily the carotid body (Iturriaga et al. 2021), acutely upregulating respiration. In conditions of low PaO_2_, type I (glomus) cells in the carotid bodies signal specialized brainstem nuclei to elicit an autonomic nervous system response that increases ventilation, sympathetic activation, heart rate. and arterial blood pressure which, when combined with peripheral vasodilation but pulmonary vasoconstriction, serve to improve oxygen supply to tissues. However, these responses also may produce pathophysiological effects resulting in altitude illnesses.

More specifically, the carotid body senses PaO_2_, the partial pressure of CO_2_ (PaCO_2_) and pH in the arterial blood, initiating reflex responses to maintain cardiorespiratory homeostasis (Iturriaga et al. 2021; Iturriaga and Diaz 2025). The carotid body consists of clusters of type I cells arranged around the capillaries and enclosed by type II cells. The type I cells make synaptic contact with the nerve terminals of petrosal afferent neurons that project to the nucleus of the tractus solitarius (NTS).

According to the current model of transduction, hypoxia induces the inhibition of voltage‐gated K^+^ (KvO_2_) and background (TASK) K^+^ channels, leading to type I cell depolarization, entry of Ca^2+^ and release of adenosine triphosphate (ATP) and acetylcholine, which increase the rate of discharge in the afferent neurons (Iturriaga et al. 2021). Hypoxia‐induced regulation of mitochondrial oxidative phosphorylation and associated ROS production (Gao et al. 2022), and ATP depletion (Varas et al. 2007) has been suggested to control the opening of KvO_2_ and TASK K^+^ channels, respectively, eliciting membrane depolarization, entry of Ca^2+^ and release of excitatory transmitters.

Increasing evidence implicates the carotid body in resistant hypertension, congestive heart failure, obstructive sleep apnea, and metabolic disorders (Iturriaga 2023; Żera et al. 2025). Increased carotid body chemosensory sensitivity and reactivity to hypoxia are linked to sympathetic hyperactivity, a common hallmark of these human diseases. The mechanisms behind carotid body chemosensory potentiation remain incompletely understood, but oxidative stress and inflammation appear to be involved. Ablating carotid body chemosensory input to the NTS reduces sympathetic overactivity and cardiorespiratory changes in preclinical models of severe hypertension and OSA in rats and mice (Pereyra et al. 2025; Żera et al. 2025). Accordingly, a comprehensive understanding of the mechanisms underlying carotid body chemosensing in humans is essential for translational research and clinical applications.

Most understanding of human carotid body chemosensing is based on ventilatory responses to hypoxia. The importance of carotid body chemoreception has been shown by ablation and denervation studies. Nakayama used uni‐ or bilateral carotid body resections to reduce air hunger in many bronchial asthma patients (Nakayama 1961). He observed that patients with severe asthma experienced some improvement 2 years after carotid body resection. Honda and colleagues studied the HVR two decades after surgery, demonstrating that bilateral carotid body ablation abolished the reflex response to hypoxia and significantly compromised CO_2_ chemosensitivity. Consequently, bilateral resection of the carotid bodies in humans led to a permanent loss of HVR under normocapnic conditions (Honda et al. 1979). The carotid bodies in humans thus are thought to be the most important components controlling the prominent ventilatory and cardiovascular responses to hypoxia. In contrast, a partial recovery of the HVR was reported after carotid body denervation in rodents, suggesting an involvement of central oxygen‐sensitive elements that can induce ventilatory hypoxia responses in these species independently of the carotid body (Teppema and Dahan 2010; Gourine and Funk 2017).

Most of the available physiological and morphological information about human carotid bodies has been obtained from autopsy material. The structures of the human and rodent carotid bodies are similar. The mean carotid body size in humans is ∼2–3 mm, with a mean weight of ∼20 mg (Heath 1991; Ortega‐Sáenz et al. 2013), although large variation in shapes has been noted (Khan et al. 1988). A high plasticity of the carotid body further increases the inter‐human variability (Kumar and Prabhakar 2012). In rats, the carotid body is smaller in proportion to the smaller mass of rat versus human: ∼1 mm in size, it usually weighs 1–3 mg and contains ∼12,000 type I cells (McDonald and Larue 1983; Atanasova et al. 2011). Structure and size can vary substantially among rat strains; for example, the carotid body of normotensive Wistar rats is one‐third that of hypertensive Okamoto rats (Smith et al. 1984). Similarly, carotid body volume varies appreciably among mouse strains, averaging about 6.3 × 10^6^ μm^3^ in DBA/2 J mice versus 1.5 × 10^6^ μm^3^ in A/J mice (Yamaguchi et al. 2003). Most carotid body chemosensing studies were performed in mice and rats, with limited data available on humans. However, Ortega‐Sáenz et al. (2013) using carotid bodies from human donors, demonstrated that the cellular properties of the human CB chemoreceptor cells resemble those of rats and mice. Thus, the type I cells of all three species respond to hypoxia and hypoglycemia by increasing the cytosolic Ca^2+^ and releasing catecholamines.

Taken together, the available evidence indicates that the physiological functions of the carotid body are comparable in humans, rats and mice. Although this interspecies concordance supports studies in rodents for the development of novel translational therapies for autonomic and cardiorespiratory disorders, the apparently stronger reliance of the HVR on the carotid body in humans versus rodents, and differential respiratory responses to hypoxia must be taken into account.

### 4.4 Respiration and Pulmonary Gas Exchange

Despite the broad similarities of cardiorespiratory physiology among mammals (Gonzalez and Kuwahira 2018), there are some important differences between humans and rodents. Functional pulmonary variables, such as tidal volume, vital capacity and total lung capacity, are closely related to the body mass of individual species; thus, the scaling of these variables versus body mass is close to unity in humans, rats and mice (Gonzalez and Kuwahira 2018). In contrast, mass‐adjusted minute ventilation (V̇E) is greater in smaller mammals; consequently, V̇E/unit mass is much higher in mice and rats than in humans. This V̇E pattern roughly parallels oxygen consumption (V̇O_2_), so V̇E/V̇O_2_ ratios are similar across species (Gonzalez and Kuwahira 2018). The larger surface‐to‐volume ratio of smaller animals increases heat loss, necessitating higher metabolic heat production to compensate (Gonzalez and Kuwahira 2018). Despite the quantitative differences among species, the V̇E versus carbon dioxide output relationship (V̇E/V̇CO_2_) and arterial or peripheral oxygen saturation (SaO_2_ or SpO_2_) are the primary factors influencing the HVR in both humans and rodents (Morgan et al. 2014).

While ventilation adapts to hypoxia in both humans and rodents, there are major quantitative and qualitative differences, for example, time courses and mechanisms of acclimatization which also may depend on age and sex (Dempsey and Forster 1982). The initial HVR is generally characterized by increases in both tidal volume and breathing frequency, but the latter's contribution is much greater in rodents than in humans (Gonzalez and Kuwahira 2018). Mice rapidly and robustly increase ventilation at hypoxia onset, but the initial response is attenuated subsequently, in a sex‐dependent manner (Palmer et al. 2013; Getsy et al. 2021). In humans, the HVR varies appreciably among individuals (Oeung et al. 2023), and, while less pronounced than in mice, lasts longer (Teppema and Dahan 2010; Getsy et al. 2021). The extant evidence indicates sex differences in the HVR are minimal in humans (Bhaumik et al. 2003), unlike mice, where appreciable sex differences are reported. Although the initial HVR was similar in male and female mice, when returned to room air, sustained elevations of ventilation were observed in males (Palmer et al. 2013). In female mice, HVR responses subsided more rapidly (“roll‐off” effect) and post‐hypoxia facilitation was less evident (Palmer et al. 2013).

Comparing mice and rats raised at sea level revealed divergent physiological responses to hypoxia which exemplified the innate capacity of mice to adapt to altitude, a capacity that humans and rats largely lack (Jochmans‐Lemoine et al. 2016). Rats have indeed long been considered an excellent model of human ventilatory adaptation to hypoxia (Olson Jr. and Dempsey 1978). Of the two rodent species, mice exhibit more pronounced ventilatory and metabolic responses to acute hypoxia, while rats show faster and larger hematological responses (Arias‐Reyes et al. 2021). These differences may explain why mice tolerate high‐altitude exposure (i.e., severe hypoxia) better than rats (and humans). Comparing ventilatory responses with increasing hypoxia, Gonzalez and Kuwahira (2018) confirmed the higher ventilatory response in mice versus rats, although both rodent species showed a much steeper increase in ventilation than humans when inspired oxygen fractions decrease.

Similar to humans, acute high‐altitude/hypoxia exposure of rats initiates autonomic and baroreflex control by provoking parasympathetic withdrawal (Beltrán et al. 2020; Alvarez‐Araos et al. 2024). In humans, acute hypoxia resets the arterial baroreflex, leading to protracted sympathetic nervous system activation, that is, sympathetic long‐term facilitation (Shafer et al. 2022). Changes in sympathetic–respiratory coupling and sympathetic overactivation result in systemic hypertension when rats (and likely also humans) are acutely exposed to sustained hypoxia. By contrast, under these conditions mice exhibit little if any increase in blood pressure and a reduction in heart rate despite elevated breathing frequency (Rodrigues et al. 2021).

Collectively, these studies demonstrate that acute hypoxia robustly stimulates respiration via the peripheral chemoreceptors in both mice and rats (Jochmans‐Lemoine et al. 2016), but the systemic responses to hypoxia appear to be fundamentally different in these species. Thus, hypoxia elicits distinct physiological, anatomic‐structural, and molecular adaptations in rats versus mice (Jochmans‐Lemoine et al. 2018).

### 4.5 Cardiovascular Responses and Hypoxic Hypometabolism

**FIGURE 2:**
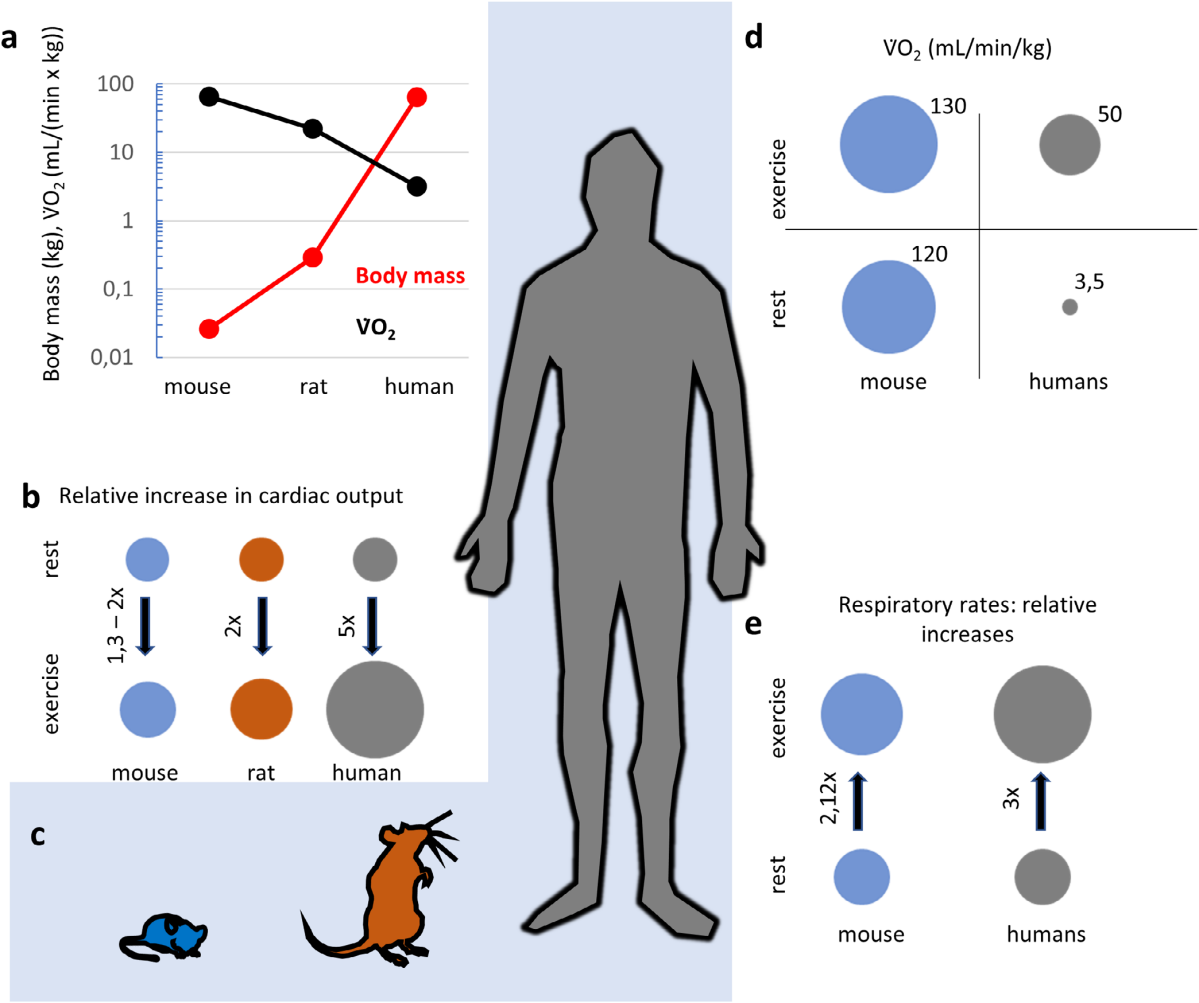
Exercise‐related differences in cardiorespiratory physiology between humans, rats and mice. (a) Mass‐normalized oxygen uptake (V̇O_2_) decreases with increasing body mass. (b) Humans increase cardiac output much more than rodents. (c) Approximate size relations between humans, rats and mice. (d) From rest to maximal exercise, V̇O_2_ increases only by about 10% in mice, versus > 10‐fold in humans. (e) Relative increases of respiratory frequency from rest to maximal exercise in mice and humans. In panels b, d and e, sizes of the circles represent relative values in mouse (blue) and human (gray).

Oxygen transport is accomplished by qualitatively similar mechanisms in humans, rats and mice. However, the enormous differences in body size and mass among these species impose quantitatively disparate requirements for oxygen import, conveyance and delivery to oxygen‐consuming tissues. Heart rates of mammalian species vary inversely with body mass, from about 1200 min^−1^ in a pygmy shrew weighing 0.004 kg, to about 6 min^−1^ in a 100,000 kg blue whale during a deep dive. Heart mass spans a similar spectrum (pygmy shrew: 0.03 g; blue whale: 500 kg). Although mammalian heart rates vary some 200‐fold, the general sequence of events comprising the cardiac cycle is conserved across all mammalian species: pacemaker depolarization, conduction and spread of depolarization, sarcoplasmic reticular Ca^2+^ release and excitation‐contraction coupling, Ca^2+^‐activated myofilament crossbridge cycling, force development and ventricular ejection, and Ca^2+^ re‐sequestration, ventricular relaxation and refilling. However, the divergent quantitative aspects of this complex process challenge extrapolation of cardiac functional measures from rodents to humans (Figure 2).

Quantitative differences in cardiac function are largely ascribable to allometric scaling, the relationship between body size and the numerical value of a quantifiable physiological variable. Allometric scaling is defined by the power equation *Y* = *α* × BM^β^, where Y is the value of a physiological variable, *α* is the coefficient for that variable applied to all species, BM is body mass, and *β* is the power exponent relating changes in the variable to changes in BM. Because the body's distributive systems, for example, the systemic vasculature, pulmonary airways and hepatobiliary tract, have fractal configurations, *β* values often approximate integer multiples of ¼ (i.e., 0.25) according to the allometric model of West et al. (1997). For example, the R‐R interval scales with a *β* approximating 0.25, the *β* value for resting heart rate is roughly −0.25, and for cardiac output *β* approximates 0.75 (Prothero 1979). Consequently, although systemic arterial systolic and diastolic pressures are similar in mice, rats and humans (Noujaim et al. 2004), resting heart rate varies inversely with body size, from 50 to 70 min^−1^ in human adults to 250–450 min^−1^ in rats and 500–700 min^−1^ in mice (Janssen et al. 2002; Lujan and DiCarlo 2013). To enable the high rates of myocardial tension development and relaxation in mice, the predominant myosin heavy chain isoform in murine myocardium, α (or MYH6) cycles roughly 5‐fold faster than the β (or MYH7) isoform, the predominant myosin heavy chain isoform in human myocardium (Rundell et al. 2005; Janssen et al. 2016).

The allometric power law for the heart rate: body mass relationship has important implications for the capacity to increase cardiac output during exercise. During intense exercise, mice can increase heart rate by about 50% at the most (e.g., from 550 to 825 bpm) (Lujan and DiCarlo 2013; Janssen et al. 2016); typically heart rate increases only by 10%–20% in exercising mice (Janssen et al. 2002) and by 27% in maximally exercising rats (Hilty et al. 1989). In contrast, humans can increase heart rate by about 300% during maximal exercise. Stroke volume also increases modestly in exercising mice, by about 15% (Lujan and DiCarlo 2013). Consequently, cardiac output increases only 1.3‐ to 2‐fold in exercising mice (Figure 2b), severely limiting murine exercise capacity. Similarly, intense exercise doubles cardiac output in rats (Hilty et al. 1989). Unlike the modest increases in exercising rodents, cardiac output may increase fivefold in maximally exercising, physically fit young adult men and women. Apart from heart rate, stroke volume and cardiac output, the responses of V̇E and V̇O_2_ to maximal exercise differ profoundly in humans versus rodents. While humans may increase V̇O_2_ from about 3.5 mL/min/kg at rest to a maximum of over 50 mL/min/kg, in mice these values increase only slightly, from about 120 mL/min/kg at rest to roughly 130 mL/min/kg during intense exercise (Petrosino et al. 2016). At best, mice can increase their respiratory frequencies from about 100 to 150 per min at rest to 180–350/min at maximal exercise (Yuki and Koutsogiannaki 2021), while in humans respiratory frequency increases by up to 300% (Figure 2e) from rest to maximal exercise (Janssen et al. 2016). Exercise performed in hypoxic conditions especially challenges the respiratory and cardiovascular systems because reduced arterial oxygen concentrations affect the function of skeletal muscles, the heart, and certain regional vascular beds, such as the pulmonary arteries (Longhurst 2003).

The minimal kinetic reserves of mice and rats limit extrapolation of cardiopulmonary responses to exercise from rodents to humans. Pulmonary gas exchange during exercise in hypoxia may be more efficient in rats than in humans, due at least in part to an increased diffusive/perfusive conductance ratio, resulting from a greater increase in pulmonary oxygen diffusion and an only modest increase in cardiac output in rats (Gonzalez and Kuwahira 2018).

Why is the capacity to increase heart rate, the cardiac “kinetic reserve” so limited in mice? Although the sizes of murine and human hearts differ dramatically, individual cardiomyocytes are nearly identical in mice and humans (Janssen et al. 2016). Consequently, in both species Ca^2+^ entering through sarcolemmal *L*‐type Ca^2+^ channels takes 10–20 ms to diffuse to the sarcomere core. Time to peak tension in human myocardium is 185 ms at rest and 120 ms during intense exercise, so Ca^2+^ entering the cardiomyocyte can participate in crossbridge activation and tension development in the early stages of systolic contraction, even at the higher heart rates provoked by maximum exertion. But in mice, the time to peak tension is only 35 ms even at rest. Whether the mouse is resting or exercising, the Ca^2+^ entering the cardiomyocyte cannot diffuse to the sarcomere core quickly enough to activate crossbridge cycling and tension development. Consequently, murine cardiomyocytes rely almost entirely on Ca^2+^ released by the sarcoplasmic reticulum for excitation‐contraction coupling, while only 3% of the crossbridge‐activating Ca^2+^ enters through the sarcolemma, versus roughly 30% in human cardiomyocytes. Sarcolemmal Ca^2+^ entry produces a distinct “plateau phase” in the human cardiomyocyte action potential, while the action potential plateau is nearly absent in murine cardiomyocytes (Glukhov et al. 2010).

The limited kinetic reserve in mice attenuates the force‐frequency relationship (Monasky and Janssen 2009). In humans and other species with relatively low heart rates, increased heart rate produces a marked increase in contractile force, that is, a positive force: frequency relationship, the result of increased Ca^2+^‐activation of the contractile machinery. In isolated human ventricular trabeculae, increasing stimulus frequency from 0.5 to 2.5 Hz increased tension development 81–168%, while in rat trabeculae, increasing frequency from 4 to 8 Hz increased tension only 30–40%, and in mouse trabeculae, increasing stimulus frequency from 8 to 12 Hz produced no change in tension development (Noujaim et al. 2004). Differences in sarcolemmal Ca^2+^ entry may account for the attenuated force: frequency relationships in rodents versus humans. In human cardiomyocytes, increased depolarization frequency during exercise increases time‐averaged systolic Ca^2+^ entry. During diastole, the sarcoplasmic reticulum sequesters some of this extra Ca^2+^, increasing reticular Ca^2+^ loading and, thereby, systolic Ca^2+^ release, producing a more forceful contraction. Because sarcolemmal Ca^2+^ entry is limited in rats and negligible in mice, the force: frequency relationship is comparatively modest in rats, and essentially absent in mice.

Mice and rats are facile, cost‐effective tools to investigate mechanisms of cardiovascular physiology and pathophysiology, and extensive research in these small mammals has advanced understanding of cardiac mechanics and the impact of cardiometabolic disease on the heart. Nevertheless, the important distinctions in the physiology of rodent versus human myocardium must be kept in mind when extrapolating outcomes of rodent studies to the human cardiovascular system. This limitation may be particularly relevant for studies in hypoxia, especially when considering the divergent metabolic responses in humans and rodents.

A striking difference between humans and rodents is the transitory and temperature‐dependent decrease of metabolism in rodents, first termed “hypoxic hypometabolism” by Mortala and colleagues in the 1990s (Mortola 1993) that develops within several minutes of hypoxia exposure. Mediated by the hypothalamus (Gu and Jun 2018), this phenomenon, which does not occur in human adults (Gonzalez and Kuwahira 2018; Gu and Jun 2018; Joyce and Wang 2022), is characterized by a decrease of metabolic rate and an increase of the V̇E/V̇O_2_ ratio, due to increased minute ventilation and reduced oxygen uptake (Gonzalez and Kuwahira 2018). Hypoxic hypometabolism is further associated with, and might be partially ascribable to, reduced body temperature, attenuated shivering/non‐shivering thermogenesis, peripheral vasoconstriction and reduced heart rates. Mice, despite initially displaying tachycardic responses to acute hypoxia, after about 10–15 min develop bradycardia lasting for several hours (Joyce and Wang 2022). Although hypoxic hypometabolism appears to be much less pronounced in rats (Jochmans‐Lemoine et al. 2015), it has been reported (Mortola and Seifert 2000) also in larger rodents especially in cold conditions, and serves to adjust the metabolism to reduced oxygen availability. In contrast, humans respond to hypoxic conditions by increasing heart rate and accelerating anaerobic energy metabolism, a response that likely underlies the increased glucose turnover observed at high altitude (Roberts et al. 1996). While there are indications that hypoxia impairs thermoregulation in humans, this impairment is not related to hypoxic hypometabolism (Gu and Jun 2018).

The cardiovascular system of rats may be particularly vulnerable to hypoxia, as indicated by severe myocardial injury and electrocardiogram abnormalities in Sprague Dawley rats exposed for several days to a simulated altitude corresponding to 6000 m (Xie et al. 2021; Wang et al. 2024). These untoward effects, termed “high‐altitude myocardial injury” appear to be specific to rats, since no such pathologies usually occur in mice or in humans, especially those without pre‐existing cardiovascular pathologies (Burtscher and Burtscher 2024).

In summary, species‐specific cardiovascular responses importantly influence human, rat and mouse strategies to overcome physiological challenges, such as exercise and/or hypoxia. The striking differences in cardiovascular reserve capacities and in acute metabolic responses to hypoxia among these species underscore the need for caution when generalizing results from rodents to humans.

### 4.6 Hematological Parameters and Oxygen Affinity

**FIGURE 3:**
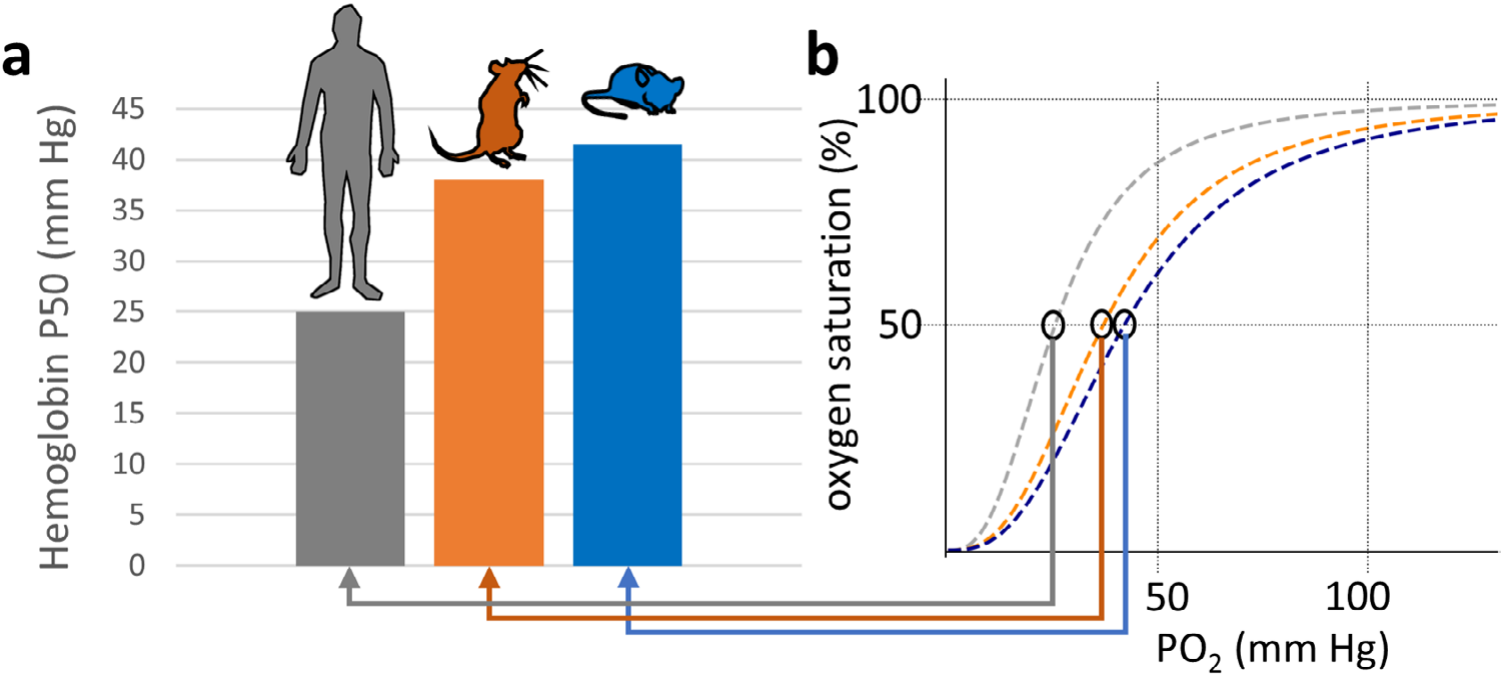
Oxygen binding of hemoglobin in humans, rats and mice. (a) Hemoglobin P50 values are depicted: The partial pressure of oxygen (PO_2_) at which the saturation of hemoglobin with oxygen is 50%. (b) Estimations of the oxygen hemoglobin curves for the 3 species based on the values published by Gray and Steadman (1964).

Translating data from rodents to humans is complicated also by lifespan variations and associated temporal differences in development and maturation. For example, the lifespan of erythrocytes is 60 days in mice versus 120 days in humans (Bogdanova et al. 2007). The oxygen affinity of hemoglobin also differs between species (Figure 3). As Gray and Steadman (1964) reported over 60 years ago, hemoglobins of mice and rats have much lower oxygen affinities compared to human hemoglobin, allowing a higher oxygen extraction rate in these animals (Gonzalez and Kuwahira 2018). The hemoglobin P50 values (the partial pressure of oxygen at which the oxygen saturation of hemoglobin is 50%) are approximately 25, 38 and 41.5 mmHg in human, rat and mouse blood, respectively (Gray and Steadman 1964). Moreover, human erythrocytes have larger mean corpuscular volumes and lower 2,3‐diphosphoglycerate concentrations than rodents (An et al. 2015). Of note, the arterio‐venous oxygen saturation difference is lowest in humans and highest in mice.

Substantial decreases of arterial oxygen content in humans typically occur at altitudes above 2500 m (Moore 2017). However, in humans, hematological adaptations are approximately linear with increasing altitude already from low altitude locations (Gassmann et al. 2019; Staub et al. 2020). These hematological adaptations strikingly differ in mice versus rats, with pronounced increases of hemoglobin and hematocrit at high altitude (3600 m) in rats, but little change in mice (Jochmans‐Lemoine et al. 2015; Arias‐Reyes et al. 2021). In contrast, mice showed a massively increased ventilation at high altitude, which was not observed in rats (Jochmans‐Lemoine et al. 2015; Arias‐Reyes et al. 2021). These divergent responses in rodents are intriguingly reminiscent of the differences in physiologic adaptations in human high‐altitude populations on the Tibetan plateau and in the Andes. While Tibetan highlanders are characterized by a higher resting ventilation and no elevated hemoglobin concentration, the opposite is true for most Andean highlanders (Bigham and Lee 2014), who suffer more frequently from chronic mountain sickness. However, an analysis of minute ventilation in humans, rats and mice showed a still stronger early ventilatory response of rats than humans acutely exposed to hypoxia (Gonzalez and Kuwahira 2018).

## 5 Conclusions

Prolonged hypoxia—continuous or intermittent—can confer health benefits. Notably, the most compelling evidence of the therapeutic efficacy of intermittent or continuous hypoxia originates from animal models, particularly mice. In these models, protocols have been identified that produce the desired effects. Whether these findings translate to humans remains uncertain, and direct cross‐species comparisons of hypoxia responses and adaptations are surprisingly scarce. Such comparisons are essential for safely advancing hypoxia‐based therapies to patient care.

Although the basic oxygen uptake, sensing and transport parameters are qualitatively (but not quantitatively) similar in humans and rodents, several responses to hypoxia are markedly different. While humans maintain their metabolic rate in hypoxia by increasing ventilation, oxygen transport and anaerobic metabolism, rodents, especially mice, employ a strikingly different strategy during the initial stages of hypoxic adaptation: they lower their metabolic rate. This metabolic downregulation is notably associated with hypothermia and reduced heart rate (following an initial tachycardia during the first few minutes of acute hypoxia). Although hypoxic chemosensing mechanisms in humans, rats and mice all induce hyperventilation, there are significant temporal and quantitative differences. Furthermore, the contribution of central elements regulating cardiorespiratory hypoxic responses independently of carotid bodies varies between species and is still a matter of debate. Indeed, unlike humans, rodents appear to partially compensate for the loss of peripheral chemoreceptor‐mediated HVR after carotid body denervation.

Anatomical, physiological, and cellular distinctions further impede direct extrapolation. When exercise in hypoxia is studied, large interspecies differences in the capacity to augment V̇E, V̇O_2_, and cardiac output—and the correspondingly limited cardiac “kinetic reserve” in rodents—must be considered. Species‐specific immune, inflammatory, and redox programs also diverge: while intermittent and moderate chronic hypoxia often improve oxidative‐stress and inflammatory control, the magnitude and direction of these effects can differ markedly between humans, rats, and mice.

Overall, in line with their phylogeographical history, mice exhibit exceptional high‐altitude tolerance—exceeding that of humans—likely driven by higher mass‐corrected lung volume and alveolar surface area, more efficient pulmonary gas exchange, hypoxic hypometabolism, and greater mitochondrial electron‐transport plasticity. In contrast, high‐altitude myocardial injury in rats—not seen in healthy humans or mice—and the more pronounced pulmonary vascular remodeling response to hypoxia in rats versus mice indicate that rats are particularly vulnerable to hypoxia. This vulnerability is further supported by the observation of greater right ventricular hypertrophy and excessive erythropoiesis in laboratory rats, but not mice, housed at high altitude.

Like all animal models, rodent models have advantages and disadvantages. They are useful to study specific aspects of hypoxia responses; however, the highlighted species differences and potentially small translational validity must be considered. Larger animals—like pigs or non‐human primates—may reflect certain aspects of human hypoxia adaptations (especially quantitatively) better than rodents, but they have ethical, economic and logistical (more challenging to expose to hypoxia) limitations, reducing their value as models for hypoxia research.

**FIGURE 4:**
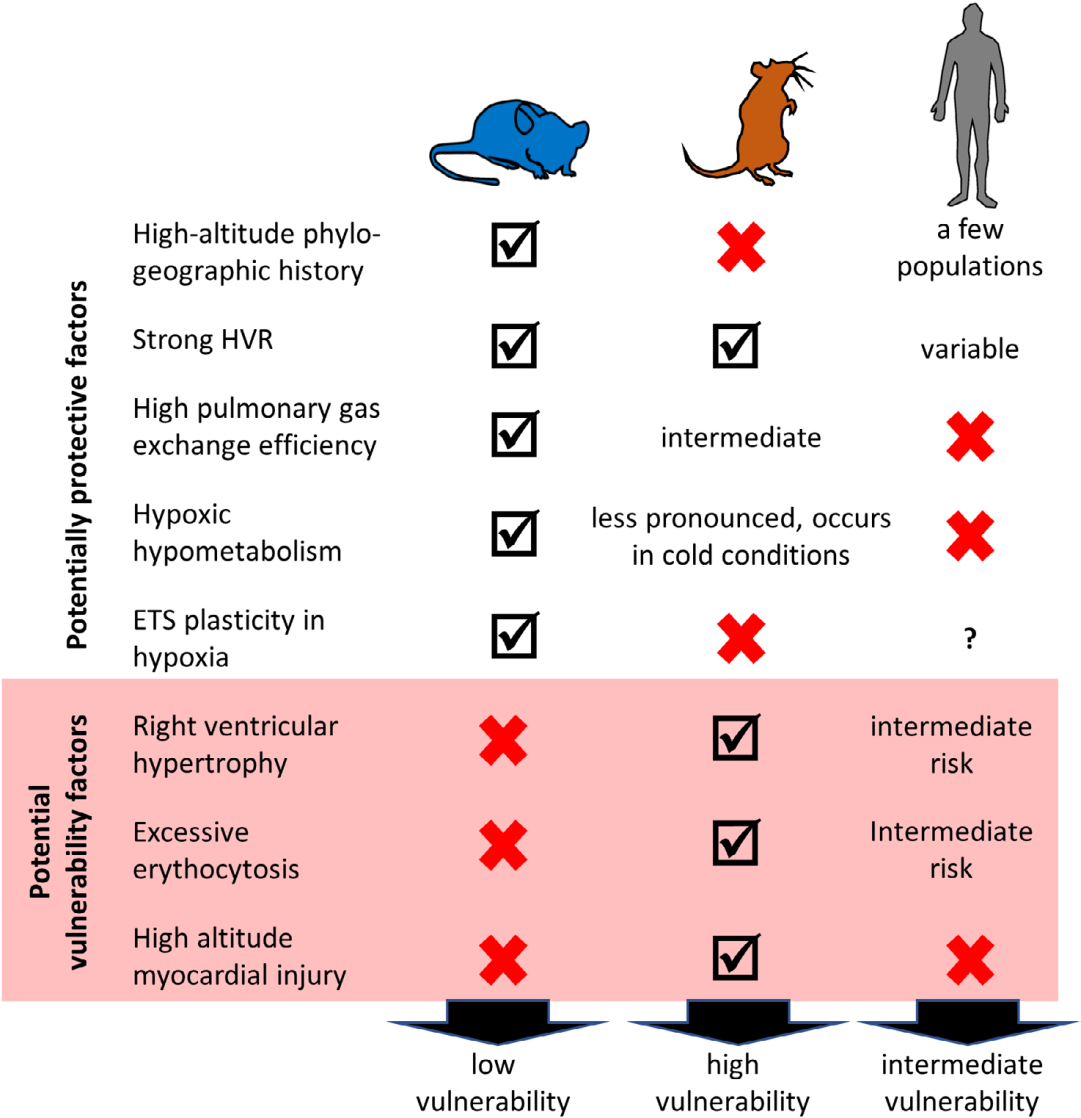
Comparison of protective and detrimental factors for hypoxia/altitude tolerance. ETS: (mitochondrial) electron transport system, HVR: Hypoxic ventilatory response.

Therefore, no single “ideal” animal model for pre‐clinical hypoxia research exists; but combining studies in mice and rats with studies in large animal models can provide additional information on mechanisms, safety and practicality of potential treatments, permitting eventual translation of research discoveries to humans. Although generally good safety profiles often allow studying therapeutic hypoxia applications directly in humans as well, mechanistic studies are more difficult to perform in humans (e.g., limited access to tissues, ease of genetic manipulation especially in mice). Technological advances (imaging, simulation and analytical techniques, etc.) will increasingly overcome these limitations. Moreover, advanced cell‐culturing methods—such as induced Pluripotent Stem Cells‐derived organoids, tissue slices and microfluidic ‘organ‐on‐chip’ platforms—allow the in vitro study of hypoxia responses of specific cell types in close‐to‐physiological environments, also considering inter‐cell/tissue interactions and inter‐individual variability. Figure 4 compares the protective and detrimental factors impacting hypoxia/altitude tolerance in humans, rats and mice.

Taken together, extrapolation of the results of rodent studies on hypoxia interventions to humans requires great caution. The divergent responses of humans, rats and mice to hypoxia are neither fully characterized nor understood, and remain important topics for preclinical and clinical investigation. Recommendations for translational hypoxia research are provided in Box 1.

BOX 1 **Recommendations for Translational Hypoxia.**

*Immediate implications (what labs could change now)*

— Explicit “translational fit” statements should be added to rodent studies.
— Dosing windows of intermittent/continuous hypoxia protocols must be considered; what is “therapeutic” in mice may be harmful or impractical in humans; what is harmful in rats may be therapeutic in humans.

*Near‐term research directions*

— A new focus on comparative physiology and molecular biology is required to better understand the validity of animal models, especially – but not only – for hypoxia research.
— Development of valid in vitro systems for systemically and physiologically relevant hypoxia responses.
— Biomarkers: cross‐species panels (e.g., ventilatory equivalents, lactate dynamics, EPO/hemoglobin mass, RV strain, cytokine/redox indices) should be developed to enable back‐translation.

*Translation and clinical development*

— Prioritization of human cohorts for research on clinical applications of hypoxia interventions.

## Author Contributions

J.B. conceptualized the article, wrote the first draft and prepared the visualizations. R.T.M., A.S., M.G., M.B. and R.I. wrote the review. All authors have read and approved the final version of this manuscript and agree to be accountable for all aspects of the work in ensuring that questions related to the accuracy or integrity of any part of the work are appropriately investigated and resolved. All persons designated as authors qualify for authorship, and all those who qualify for authorship are listed.

## Funding

The authors have nothing to report.

## Conflicts of Interest

The authors declare no conflicts of interest.

## Data Availability

No data have been generated for this review.
